# A diffusion-matched principal component analysis (DM-PCA) based two-channel denoising procedure for high-resolution diffusion-weighted MRI

**DOI:** 10.1371/journal.pone.0195952

**Published:** 2018-04-25

**Authors:** Nan-kuei Chen, Hing-Chiu Chang, Ali Bilgin, Adam Bernstein, Theodore P. Trouard

**Affiliations:** 1 Department of Biomedical Engineering, University of Arizona, Tucson, Arizona, United States of America; 2 Department of Medical Imaging, University of Arizona, Tucson, Arizona, United States of America; 3 Brain Imaging and Analysis Center, Duke University Medical Center, Durham, North Carolina, United States of America; 4 Department of Diagnostic Radiology, The University of Hong Kong, Hong Kong, Hong Kong; 5 Department of Electrical and Computer Engineering, University of Arizona, Tucson, Arizona, United States of America; 6 BIO5 Institute, University of Arizona, Tucson, Arizona, United States of America; 7 Evelyn F McKnight Brain Institute, University of Arizona, Tucson, Arizona, United States of America; University of North Carolina at Chapel Hill, UNITED STATES

## Abstract

Over the past several years, significant efforts have been made to improve the spatial resolution of diffusion-weighted imaging (DWI), aiming at better detecting subtle lesions and more reliably resolving white-matter fiber tracts. A major concern with high-resolution DWI is the limited signal-to-noise ratio (SNR), which may significantly offset the advantages of high spatial resolution. Although the SNR of DWI data can be improved by denoising in post-processing, existing denoising procedures may potentially reduce the anatomic resolvability of high-resolution imaging data. Additionally, non-Gaussian noise induced signal bias in low-SNR DWI data may not always be corrected with existing denoising approaches. Here we report an improved denoising procedure, termed diffusion-matched principal component analysis (DM-PCA), which comprises 1) identifying a group of (not necessarily neighboring) voxels that demonstrate very similar magnitude signal variation patterns along the diffusion dimension, 2) correcting low-frequency phase variations in complex-valued DWI data, 3) performing PCA along the diffusion dimension for real- and imaginary-components (in two separate channels) of phase-corrected DWI voxels with matched diffusion properties, 4) suppressing the noisy PCA components in real- and imaginary-components, separately, of phase-corrected DWI data, and 5) combining real- and imaginary-components of denoised DWI data. Our data show that the new two-channel (i.e., for real- and imaginary-components) DM-PCA denoising procedure performs reliably without noticeably compromising anatomic resolvability. Non-Gaussian noise induced signal bias could also be reduced with the new denoising method. The DM-PCA based denoising procedure should prove highly valuable for high-resolution DWI studies in research and clinical uses.

## 1. Introduction

Diffusion weighted imaging (DWI), through characterizing properties of proton diffusion, can assess microstructural changes of brain tissues resulting from neurological diseases [1, 2]. Additionally, DWI data that are appropriately sampled in q-space can be fit to a tensor or other models to characterize the structure of white-matter. For example, diffusion tensor imaging (DTI) has proven to be a valuable tool for mapping the structural connectivity networks of brains [3–8].

In the past few years, significant efforts have been made to improve the spatial resolution of DWI, aiming at better detection of subtle brain lesions and more reliably resolving white-matter fiber tracts. Specifically, parallel MRI techniques [9–11] and multi-shot diffusion-weighted MRI procedures that can correct for shot-to-shot phase inconsistencies are the keys in enabling high-resolution DWI [12–42]. The advantages of high-resolution DWI have been demonstrated in a series of recent reports [18,43–45].

A concern in high-resolution DWI is the limited signal-to-noise ratio (SNR). The SNR of DWI data obtained with conventional protocols (e.g., with 2 x 2 x 2 mm^3^ voxel size for human MRI) is already lower than non-DWI data as a result of diffusion-weighting, and is further reduced in high-resolution scans due to voxel size reduction (e.g., by 8-fold in human MRI data of 1 x 1 x 1 mm^3^ resolution). Such reductions in SNR can significantly offset the advantages of high-resolution DWI. Furthermore, non-Gaussian noise induced signal bias may distort the quantitative measures (e.g., apparent diffusion coefficient (ADC)) derived from DWI data of low SNR.

Several post-processing methods can be used to improve the SNR of high-resolution DWI data. For example, DWI data with low SNR can be denoised with a kernel smoothing procedure. However, the anatomic resolvability of high-resolution DWI data may be significantly compromised by kernel smoothing. This concern can be partially addressed with the recently reported local principal component analysis (local PCA) algorithm [46], which could effectively reduce noise through filtering data with PCA along the diffusion dimension (i.e., the q-dimension). However, the local PCA method may still potentially reduce anatomic resolvability, because it performs PCA filtering across nearest neighboring voxels that may have heterogeneous diffusion properties. With a highly heterogeneous set of neighboring voxels (that have a low redundancy in the contained information), the performance of PCA filtering may degrade for DWI data with a small number of diffusion-encoding directions (e.g., 3 diffusion directions in most clinical DWI protocols).

Previous studies have shown that Rician signal bias could be estimated from background areas, and then subtracted from low-SNR data [47,48]. However, in DWI data obtained with parallel MRI, the reconstructed magnitude signals may be affected by other types of spatially-dependent and non-Gaussian noise, which may not always be correctable with existing post-processing procedures. A recent report by Eichner et al. showed that non-Gaussian noise in parallel DWI data could be better reduced by averaging multiple sets of phase-corrected and real-valued data [49]. However, Eichner’s noise reduction scheme requires repeated scans of multiple averages.

In this paper we report an improved denoising procedure, termed diffusion-matched principal component analysis (DM-PCA), which comprises 1) identifying a group of (not necessarily neighboring) voxels that demonstrate very similar signal variation patterns along the diffusion dimension, 2) correcting low-frequency phase variations in the complex-valued DWI data, 3) performing PCA along the diffusion dimension for real- and imaginary-components (in 2 separate channels) of phase-corrected DWI voxels with matched diffusion properties, 4) suppressing the noisy PCA components in real- and imaginary-components, separately, of phase-corrected DWI data, and 5) combining real- and imaginary-components of denoised DWI data. Inspired by the collaborative filtering algorithm reported by Dabov et al. [50], our method is designed to perform PCA filtering across a group of voxels that have a high level of redundant information in order to preserve anatomic resolvability. It should be noted that our method performs PCA along the diffusion dimension and thus differs from Dabov’s original implementation. Additionally, the non-Gaussian noise induced signal bias could be reduced with our new approach that performs PCA on complex-valued DWI data.

## 2. Theory

MRI data were acquired with a 3 Tesla MRI scanner (General Electric Healthcare, Waukesha, USA) at Duke University Medical Center. All research involving human participants has been approved by the Institutional Review Board (IRB) of Duke University Medical Center. Written and oral informed consents have been obtained from all the participants. All of the de-identified MRI data are available from this public repository: https://dataverse.harvard.edu/dataverse/PONE-D-17-40504R1 and https://doi.org/10.7910/DVN/JVSBC7

### 2.1 The diffusion-matched principal component analysis based denoising procedure

#### 2.1.1. Overview

**Fig 1:**
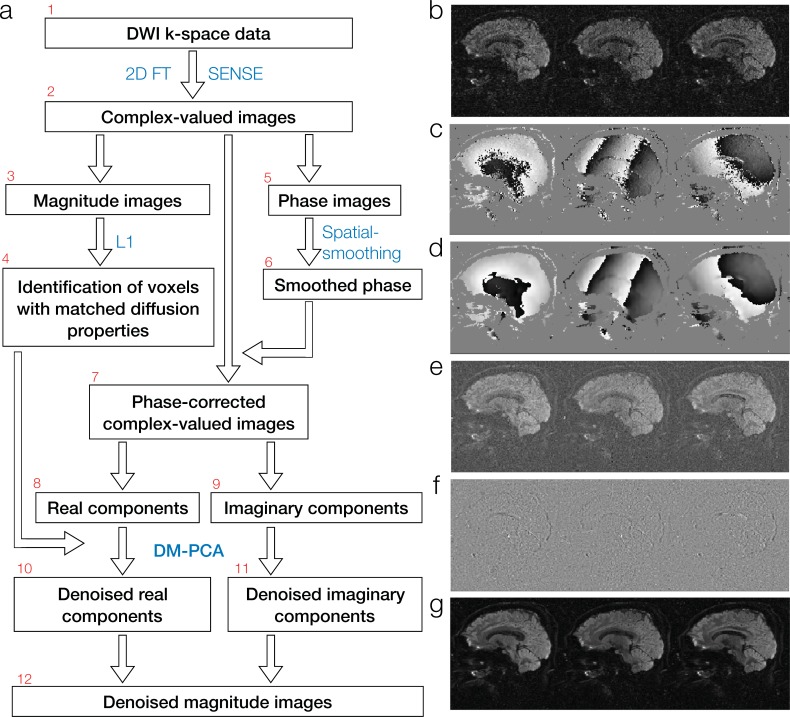
The schematic diagram of the new complex-domain DM-PCA based two-channel denoising procedure.

The new denoising method is schematically illustrated in Fig 1A. Depending on the acquisition protocols, the acquired DWI k-space data (box 1) are reconstructed with either 2D Fourier transform or parallel MRI procedures (such as the SENSE algorithm [9]) to generate complex-valued images (box 2). For any given voxel to be denoised, the magnitude components (box 3 and Fig 1B) of input images are examined to identify a group of voxels with similar diffusion properties (box 4: with details described in section 2.1.2). The phase components (box 5 and Fig 1C) of input data are spatially smoothed (box 6 and Fig 1D), and the smoothed phase information is then subtracted from the input complex-valued images to produce a set of phase-corrected complex-valued images (box 7). The real-components (box 8; Fig 1E) and imaginary-components (box 9; Fig 1F) of phase-corrected data are denoised in 2 separate channels with PCA (with details described in section 2.1.3). The denoised real- and imaginary data (boxes 10 and 11, respectively) are then combined to produce a set of denoised magnitude images (box 12 and Fig 1G).

#### 2.1.2 Identification of diffusion-matched voxels

**Fig 2:**
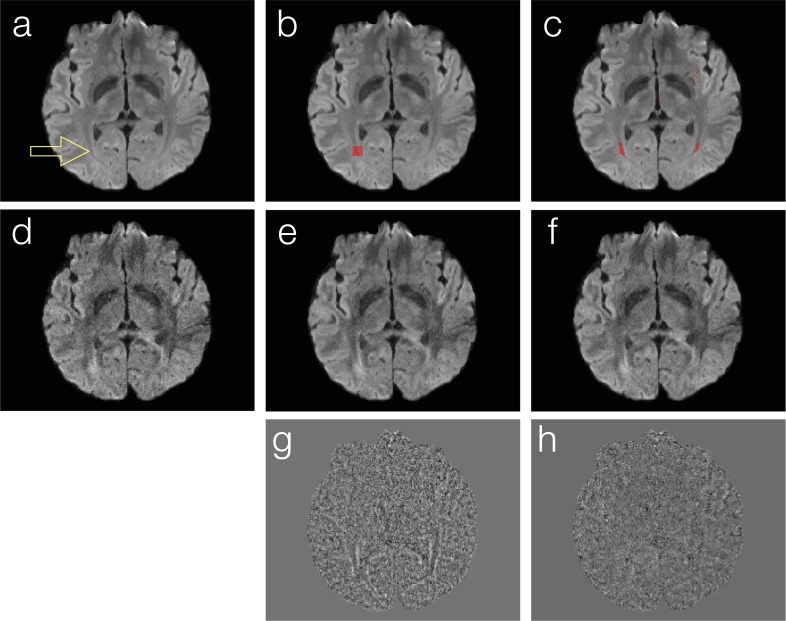
Comparison of DWI denoising through filtering signals across nearest neighboring voxels and diffusion-matched voxels: The red dot in (a) shows a target voxel (displayed on top of mean DWI map), whose signals in 6-direction DWI scans are to be denoised. In many existing denoising methods, signals of nearest neighboring voxels in a patch (see b) are the input of a filtering procedure. In contrast, we identify a group of voxels that demonstrate very similar signal variation patterns along the diffusion dimension but are not necessarily neighboring (see c) for subsequent filtering procedures. Panels d, e and f show an input image, nearest-neighboring PCA produced image, and DM-PCA produced image, respectively. Residual maps obtained with nearest-neighboring PCA and DM-PCA methods are shown in panels g and h, respectively.

An important step of the DM-PCA method is to identify a group of voxels, which have similar magnitude-valued diffusion properties (i.e., with similar signal variation patterns along the diffusion dimension) but are not necessarily nearest neighboring, from noisy input data (box 4 in Fig 1A). For example, the red dot in Fig 2A shows a target voxel (displayed on top of the mean DWI map), whose corresponding signals in 6-direction DWI data are to be denoised. Instead of filtering DWI signals of the nearest neighboring voxels in a patch (Fig 2B) as implemented in the local PCA denoising method, we use L1 norm to identify a group of voxels (Fig 2C) that have the most similar diffusion signal variations to the target voxel. Although in Fig 2B and 2C we demonstrate the selection of 100 nearest neighboring voxels (in a 10 x 10 patch) and 100 diffusion-matched voxels (across the field of view), respectively, only in a single axial slice, in our actual implementation 64 diffusion-matched voxels are identified with L1 from a 3D volume of 5 neighboring slices. We choose to identify 64 diffusion-matched voxels in processing human brain DWI data, because in our experience we could always identify more than 64 voxels located in white-matter fiber tracts that share very similar diffusion properties (e.g., red voxels in Fig 2C: with redundant diffusion information in > 64 voxels). When processing low-resolution DWI data obtained from small animals, a smaller number of diffusion-matched voxels may need to be chosen to ensure that the identified voxels have redundant diffusion information.

#### 2.1.3 Phase correction of complex-valued data

As shown in boxes 5 to 7 of Fig 1A, background phase values are smoothed and then subtracted from the input complex-valued data. After this phase correction procedure, the majority of the DWI contrast is moved to the real-components (Fig 1E). The noise in both real- and imaginary components of input data is unaffected by the removal of spatially-smooth phase values. A similar phase correction procedure was used previously by Eichner et al. for averaging repeated scans of real-valued DWI data [49]. In our project, this phase correction procedure is mainly used to generate phase-corrected complex-data for subsequent two-channel PCA denoising (see Section 2.1.5).

In our implementation, we multiplied the central 64x64 portion of the k-space data with Hann filter (of length 64: described in Eq 1), along both readout and phase-encoding directions, to smooth the background phase. We found that phase smoothing with different Hann window sizes (between 32 and 64) does not noticeable affect the final results.

$$h(n)=\frac{1}{2}(1-cos(\frac{2\pi n}{N-1})),with\;N=64;\;n=1\ldots N$$ [1]

#### 2.1.4 Comparison of nearest-neighboring PCA and diffusion-matched PCA

In this section we compare 1) images obtained from PCA among nearest-neighboring voxels and 2) data obtained from PCA among diffusion-matched voxels (i.e., DM-PCA). To simplify the discussion, here we process real-valued DWI data only. Fig 2D shows one of the 6-direction DWI input data. Using our local PCA denoising implementation (that suppresses PCA components 3 and higher across voxels shown in Fig 2B), the produced image has a higher SNR (Fig 2E) than input data. With the DM-PCA method (that suppresses PCA components 3 and higher across voxels shown in Fig 2C), the noise in input data can also be reduced (Fig 2F). Upon examining the residual signals of nearest-neighboring PCA (Fig 2G) and DM-PCA (Fig 2H), it can be seen that some anatomic features are undesirably altered in nearest-neighboring PCA produced data (likely due to the low level of redundancy in diffusion information across neighboring voxels) but not in DM-PCA produced data.

#### 2.1.5 DM-PCA for real- and imaginary components of phase-corrected data

As illustrated in boxes 8 to 11 of Fig 1A, the DM-PCA method described in section 2.1.4 is applied to denoise real- and imaginary-components, separately, of phase-corrected complex-valued data. In this section we use simulation data to illustrate the advantages of two-channel DM-PCA of complex-valued data, as compared with DM-PCA processing of magnitude images.

**Fig 3:**
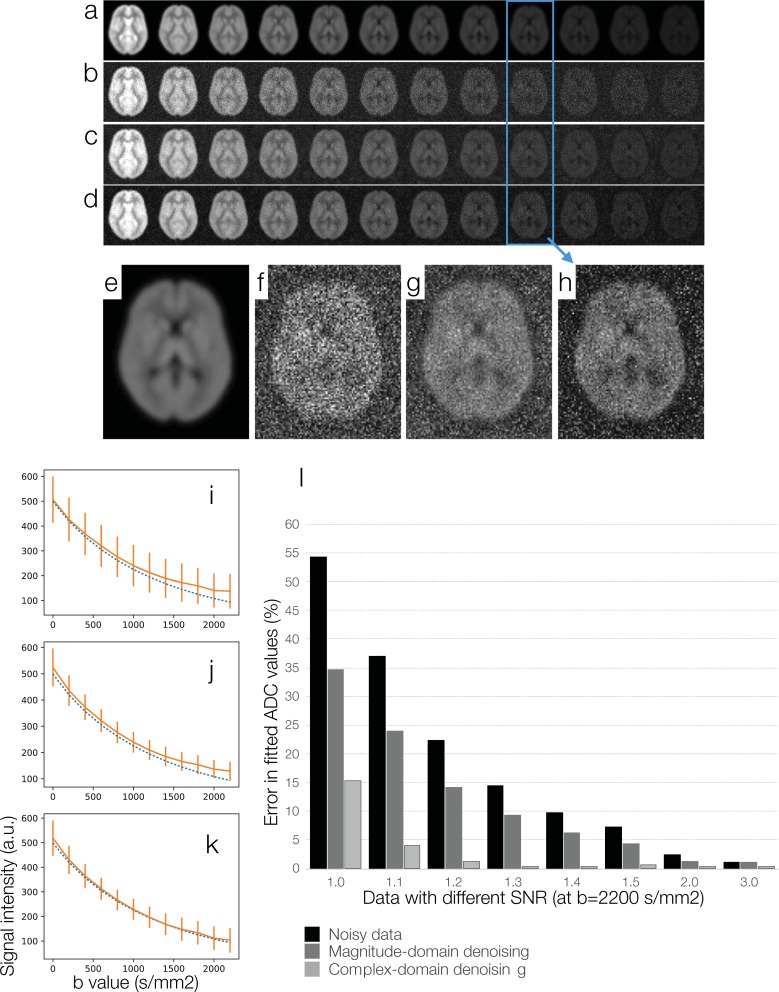
A simulation study for comparing magnitude-domain DM-PCA and two-channel complex-domain DM-PCA in terms of the accuracy in ADC fitting: (a) Noise-free DWI data corresponding to b = 0, 200, 400 … 2200 (s/mm^2^). (b) DWI data affected by Rician noise. (c) DWI data denoised by magnitude-domain DM-PCA. (d) DWI data denoised by a two-channel complex-domain DM-PCA procedure. (e) Signal intensities of noisy DWI data (solid curve in orange) and the ground truth (dashed curve in blue). (f) Signal intensities of magnitude-domain DM-PCA produced data (solid curve in orange) and the ground truth (dashed curve in blue). (g) Signal intensities of complex-domain DM-PCA produced data (solid curve in orange) and the ground truth (dashed curve in blue). (h) Errors in ADC fitting for data with different SNR levels.

A proton density image was first created by combining white-matter probability map, gray-matter probability map, and cerebrospinal fluid (CSF) probability map (from FSL: https://fsl.fmrib.ox.ac.uk) with appropriate weightings. 12 sets of noise-free DWI data corresponding to b = 0, 200, 400 … 2200 s/mm^2^ (Fig 3A) were mathematically created by multiplying the tissue-specific images with appropriate diffusion weighting, assuming that the white-matter diffusivity is 0.69⨉10^−3^ mm^2^/s, gray-matter diffusivity is 0.83⨉10^−3^ mm^2^/s, and CSF diffusivity is 3.19⨉10^−3^ mm^2^/s [51].

Full k-space data were computed from noise-free DWI data, and Gaussian noise was then added to real and imaginary components of k-space data. Images reconstructed from noisy k-space data with 2D Fourier transform thus contained noise. Specifically, real- and imaginary-components of complex-valued images contained Gaussian noise, and magnitude images contained Rician noise. Multiple data sets with different levels of noise were simulated, with the SNR of DWI at b = 2200 s/mm^2^ being 1.1, 1.2, 1.3, 1.4 … 3. One of the noisy data sets (with SNR at b = 2200 s/mm^2^ being 1.3) is shown in Fig 3B. In this simulation the levels of added noise were chosen to produce data sets with the desired SNR levels.

The noisy DWI data were denoised with two different approaches: 1) magnitude-domain DM-PCA (Fig 3C) and 2) two-channel DM-PCA (Fig 3D). Images from the blue boxes (at b = 1600 s/mm^2^) are shown in Fig 3E to 3H with an elevated display scale.

The white-matter signal intensities of noisy DWI data corresponding to different b values (Fig 3B) are shown with solid orange curve in Fig 3I, with vertical bars indicating the standard deviation across white-matter voxels. The ground truth signals (from noise-free data: Fig 3A) are shown with dashed blue curve in Fig 3I. It can be seen that the Rician bias is more pronounced in high-b data, leading to errors in the fitted ADC values.

The white-matter signal intensities of DWI data denoised by magnitude-domain DM-PCA (Fig 3C) are shown with solid orange curve in Fig 3J, and the ground truth signals are shown with dashed blue curve. It can be seen that the magnitude-domain DM-PCA could reduce the standard deviation (i.e., shorter vertical orange bars in Fig 3J than Fig 3I), but could not effectively remove the Rician bias.

The white-matter signal intensities of DWI data denoised by two-channel DM-PCA (Fig 3D) are shown with solid orange curve in Fig 3K, and the ground truth signals are shown with dashed blue curve. It can be seen that the two-channel DM-PCA method could reduce the standard deviation (i.e., shorter vertical orange bars in Fig 3K than Fig 3I), and effectively reduce the Rician bias.

Errors in fitting ADC values from DWI data, at different noise levels and with different denoising procedures, are shown in Fig 3L. It can be seen that the two-channel complex-domain DM-PCA method could more effectively reduce ADC fitting errors for all of our simulation data, as compared with magnitude-domain DM-PCA.

#### 2.1.6 DM-PCA denoising of parallel DWI data

We performed another simulation study to compare the performance of magnitude-domain DM-PCA and two-channel complex-domain DM-PCA on denoising parallel DWI data.

Similar to the study described in section 2.1.5, a proton density image was created by combining tissue-specific probability maps with appropriate weightings. Noise-free DWI data corresponding to 12 b values (0, 200, 400 … 2200 s/mm^2^) were simulated by multiplying the tissue-specific images with appropriate diffusion weighting. Shot-to-shot phase inconsistencies in DWI data were mathematically added. Afterward, the simulated DWI data were multiplied by 8 sets of coil sensitivity profiles, and converted to k-space data with 2D inverse Fourier transform. Noise was then added to real and imaginary components of 8-channel k-space data. In this simulation the levels of added noise were chosen to produce parallel DWI data with their SNR levels comparable to non-parallel DWI data shown in section 2.1.5.

**Fig 4:**
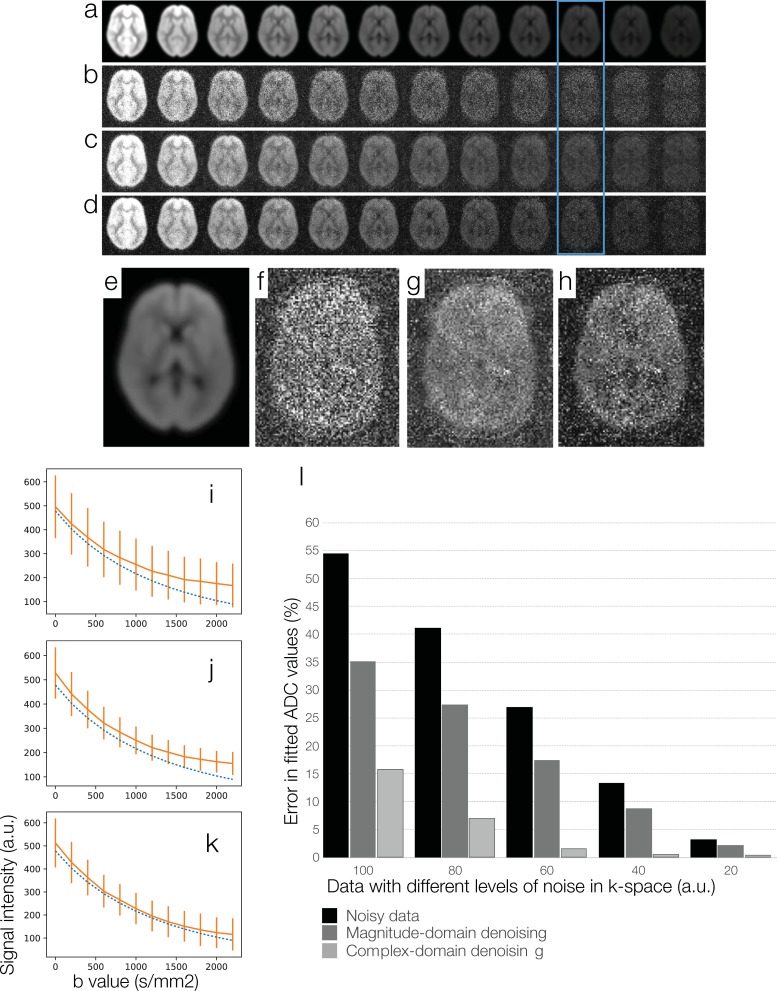
A simulation study for comparing magnitude-domain DM-PCA and two-channel complex-domain DM-PCA in terms of the accuracy in fitting ADC values from parallel DWI data: (a) Images reconstructed with the 2xSENSE algorithm from noise-free under-sampled k-space data corresponding to b = 0, 200, 400 … 2200 (s/mm^2^). (b) Images reconstructed with the 2xSENSE algorithm from noisy under-sampled k-space. (c) SENSE-produced data denoised by magnitude-domain DM-PCA. (d) SENSE-produced DWI data denoised by a two-channel complex-domain DM-PCA procedure. (e) Signal intensities of noisy parallel DWI data (solid curve in orange) and the ground truth (dashed curve in blue). (f) Signal intensities of magnitude-domain DM-PCA produced data (solid curve in orange) and the ground truth (dashed curve in blue). (g) Signal intensities of complex-domain DM-PCA produced data (solid curve in orange) and the ground truth (dashed curve in blue). (h) Errors in ADC fitting for data with different SNR levels.

Fig 4A and 4B show images reconstructed from 2x under-sampled noise-free k-space data and 2x under-sampled noisy k-space data, respectively, using the SENSE algorithm. Noise amplification due to g-factor is visible in high-b DWI data in Fig 4B.

The SENSE-produced data were then denoised with two different approaches. First, the magnitude images were processed with magnitude-domain DM-PCA. Second, the SENSE-produced complex-valued data were processed with phase-correction and the 2-channel DM-PCA denoising procedures shown in Fig 1. Fig 4C and 4D compare images denoised with the magnitude-domain DM-PCA and the two-channel complex-domain DM-PCA, respectively. Images from the blue boxes (at b = 1800 s/mm^2^) are shown in Fig 4E to h with an elevated display scale.

The white-matter signal intensities of noisy DWI data corresponding to different b values (Fig 4B) are shown with solid orange curve in Fig 4I, with vertical bars indicating the standard deviation. The ground truth signals (from noise-free data: Fig 4A) are shown with dashed blue curve in Fig 4I. The white-matter signal intensities of DWI data denoised by magnitude-domain DM-PCA (Fig 4C) are shown with solid orange curve in Fig 4J, and the white-matter signal intensities of DWI data denoised by two-channel DM-PCA (Fig 4D) are shown with solid orange curve in Fig 4K. It can be seen that the non-Gaussian noise induced bias in noisy parallel DWI data can be better removed by two-channel complex-domain DM-PCA than magnitude-domain DM-PCA.

Errors in fitting ADC values from DWI data, at different noise levels and with different denoising procedures, are shown in Fig 4L. It can be seen that the two-channel complex-domain DM-PCA method could better reduce ADC fitting errors for all of our simulation data, as compared with magnitude-domain DM-PCA.

## 3. Methods

### 3.1 Evaluation of the DM-PCA denoising method in high-resolution DWI data

The performance of the two-channel DM-PCA method was evaluated in high-resolution DWI data that were acquired previously (voxel size = 0.85 mm x 0.85 mm x 0.85 mm; 12 diffusion-encoding directions) with scan parameters reported in a recent paper [18]. The SNR of this DWI dataset was ~ 25. In order to assess the performance of DM-PCA on DWI data with different SNR levels, noise was mathematically added to the complex-valued imaging data to produce multiple additional datasets with SNR in range of 3 and 25.

DWI data before and after DM-PCA based denoising were processed with the DTI fitting program of FSL (https://fsl.fmrib.ox.ac.uk/fsl/fslwiki/FDT/UserGuide) to calculate fractional anisotropy (FA) maps. These FA maps with directionally encoded color (right-left = red; anterior-posterior = green; superior-inferior = blue) were visually inspected and compared in terms of the resolvability of adjacent fiber tracts (e.g., external capsule and extreme capsule) and small brain structures (e.g., dentate gyrus of the hippocampus).

### 3.2 Application of the DM-PCA denoising procedure to human DTI data of conventional spatial resolution

The DM-PCA denoising procedure was applied to process 60 sets of DTI data acquired from 30 healthy volunteers (age: 58.03 ± 9.28 years; 15 females) and 30 patients with Parkinson’s disease (age: 64.03 ± 10.30 years; 7 females). Twenty-five PD patients had Hoehn and Yahr scale between 1 and 2 (i.e., in early PD stages); one patient had Hoehn and Yahr scale 2.5; two patients had Hoehn and Yahr scale 3; two patients had Hoehn and Yahr scale 4. DTI data were acquired with a single-shot parallel echo-planar imaging (EPI) pulse sequence with the following scan parameters: FOV = 23cm x 23cm; in-plane matrix size = 128 x 128; parallel imaging acceleration factor = 2; TE = 80 msec; TR = 8 sec; sagittal-plane slice thickness = 1.8 mm; voxel size = 1.8 mm^3^; number of slices = 78; b = 800 s/mm^2^; number of diffusion-encoding directions = 25; number of non-DWI baseline images = 4; and number of repetitions = 2.

The SENSE algorithm was used to process the acquired under-sampled k-space data. SENSE-produced complex-valued imaging data without denoising, with magnitude-domain DM-PCA, and with two-channel complex-domain DM-PCA were all processed with the eddy current correction procedure from FSL, and then analyzed with the DTI fitting program in FSL.

FA maps from all the subjects were then registered to a common space using the skeleton based normalization provided by FSL-TBSS (https://fsl.fmrib.ox.ac.uk/fsl/fslwiki/TBSS). In this study the TBSS procedure was only used to align FA maps to a common space, and the aligned images (without being skeletonized) were further analyzed with an ROI-based analysis. Specifically, we evaluated the FA differences in the olfactory tract ROI between early-stage PD patients (Hoehn and Yahr scales 1 and 2 in 25 of our PD subjects) and controls, measured from data before and after denoising. Gender and age effects were regressed out when performing group-level analyses. This importance of measuring FA values in the olfactory tract ROI has been demonstrated in two previous reports. First, it has been shown by Ibarretxe-Bilbao et al. that FA values in the olfactory tract differ between early-stage PD patients and controls [51]. Second, studies by Rolheiser et al. [52] and Joshi et al. [53] further showed that the DTI signals of olfactory regions could better differentiate early-stage PD patients from healthy controls, as compared with signals from the substantia nigra.

## 4. Results

### 4.1 Evaluation of the DM-PCA denoising method in high-resolution DWI data

**Fig 5:**
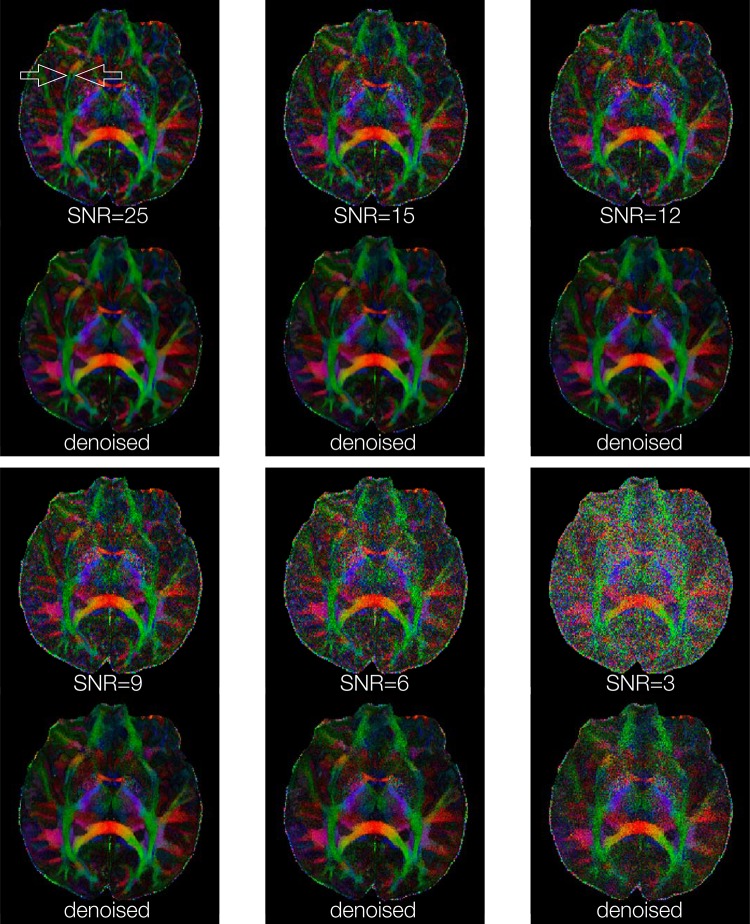
FA maps obtained from high-resolution DWI images (0.85 mm^3^ voxel size), before and after DM-PCA based denoising, corresponding to different SNR levels in input data.

Fig 5 shows FA maps, of a chosen axial slice, calculated from high-resolution DWI data (0.85 mm^3^ voxel size) with 6 SNR levels. The external capsule and extreme capsules are resolvable in the FA map calculated from the original DWI data (SNR ~ = 25) both before and after denoising. It can be seen that those two tracts remain resolvable for denoised images produced from input data with SNR = 9 or higher, demonstrating that the developed DM-PCA method does not noticeable reduce anatomic resolvability for our test datasets. For input data with low SNR (e.g., 3), the DM-PCA method may not recover high-resolution information.

**Fig 6:**
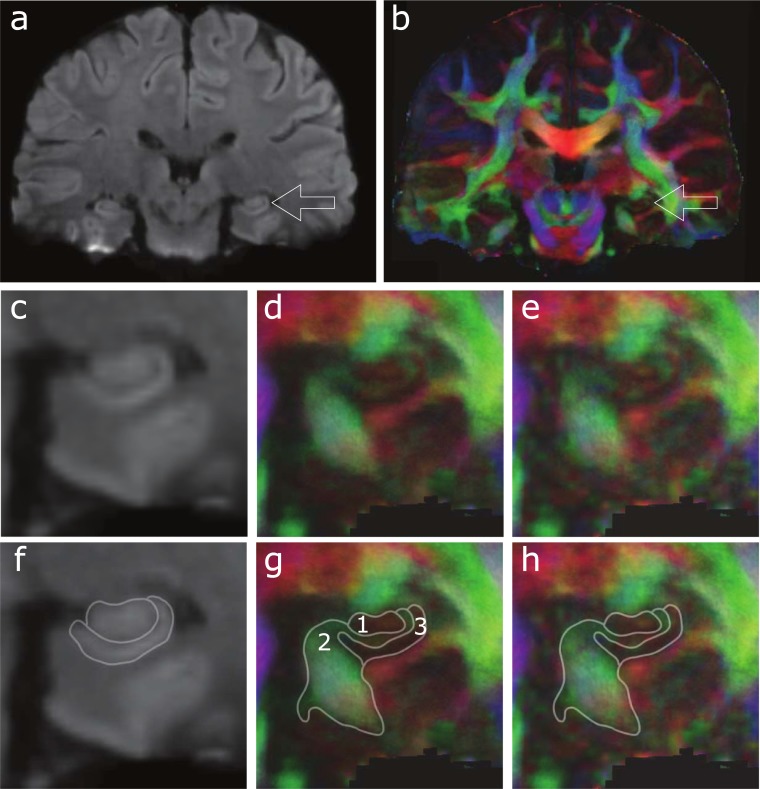
(a) and (b) show coronal-plane mean DWI and FA maps, respectively, derived from high-resolution data after DM-PCA based denoising, with arrows indicating the left hippocampus. The corresponding zoom-in images shown in (c) and (d), respectively. The FA map derived from images without DM-PCA denoising is shown in (e). The coarse hippocampal structures revealed by the mean DWI map are highlighted in (f). Anatomic structures that can be identified from color-coded FA map are shown in (g) and (h). Region 1 in (g) corresponds to the dentate gyrus; Region 2 shows fibers that connect hippocampus and entorhinal cortex to other brain areas; Region 3 contains hippocampal CA1, CA2, and CA3.

Fig 6A and 6B show coronal-plane mean DWI and FA maps, respectively, derived from high-resolution and high-SNR (~ = 25) data after DM-PCA based denoising. The hippocampus is visible in both Fig 6A and 6B (e.g., with arrows indicating the left hippocampus), as well as in the corresponding zoom-in images shown in Fig 6C and 6D, respectively. In comparison to the FA map derived from images without DM-PCA denoising (Fig 6E), the DM-PCA produced FA map (Fig 6D) preserves the anatomic features of the hippocampus. It can be seen that the mean DWI map reveals only coarse hippocampal structure (Fig 6F), while the color-coded FA map more clearly defines the dentate gyrus (region 1 in Fig 6G: with light red color indicating the connectivity to hippocampal CA3), fibers that connect hippocampus and entorhinal cortex to other brain areas (region 2 in Fig 6G: with green color indicating the connectivity along the anterior-posterior direction), and CA1, CA2 and CA3 of the hippocampus (region 3 in Fig 6G: with lower FA value).

### 4.2 Application of the DM-PCA denoising procedure to human DTI data of conventional spatial resolution

**Fig 7:**
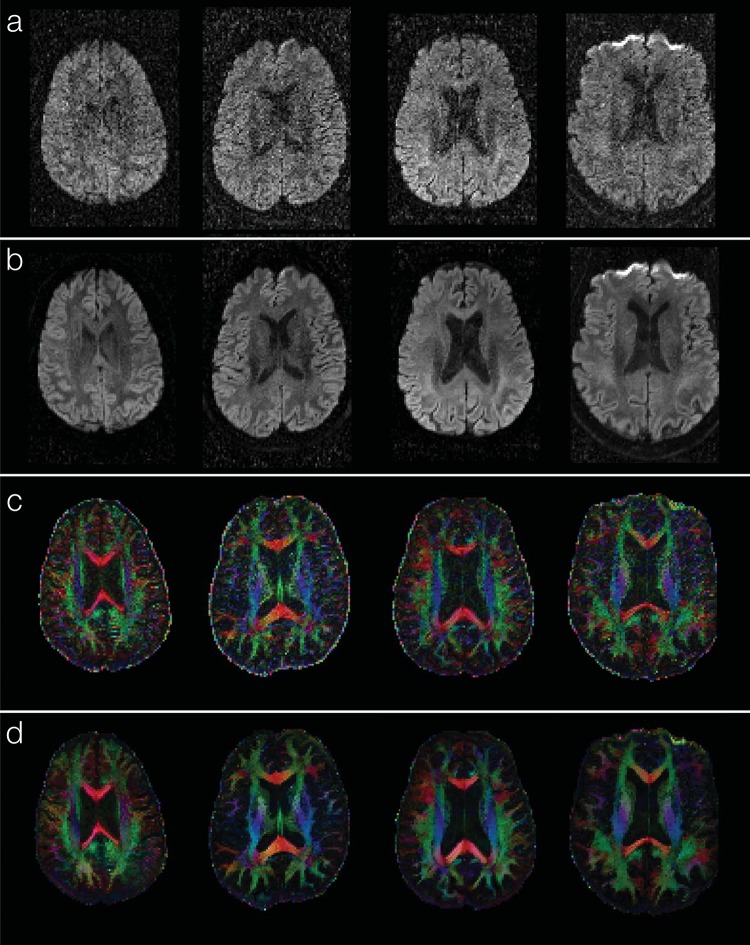
Application of DM-PCA denoising to human brain DWI data at conventional resolution (1.8 mm^3^ voxel size): Panels a and b compare one of the DWI images before and after DM-PCA denoising, respectively, for 4 of the participants. Panels c and d show the corresponding FA maps obtained from data before and after DM-PCA denoising, respectively.

Experimental results show that the new denoising method consistently improves the SNR for all of the human DWI data (1.8 mm^3^ voxel size) evaluated in this study. The sum of squared errors in DTI fitting can be reduced by ~90% for all of the denoised data, as compared with undenoised data. Fig 7A and 7B compare diffusion-weighted images (corresponding to a single encoding direction) before and after applying the DM-PCA method, respectively, for 4 of the subjects with Parkinson’s disease. Fig 7C and 7D show the corresponding FA maps before and after DM-PCA processing, respectively, in which the improvement in FA quality due to denoising can be visualized. Images denoised by magnitude-domain DM-PCA (data not shown) and two-channel complex-domain DM-PCA appear very similar through visual inspection.

In undenoised DWI data, the FA values of olfactory tract ROI are lower in PD subjects than that in healthy controls, but the difference was not statistically significant (at p = 0.08). After denoising DWI data with magnitude-domain DM-PCA, the FA measures differ significantly between PD and controls at p = 0.04. After denoising data with the two-channel complex-domain DM-PCA method, an even higher significant level, at p = 0.02, is achieved. These results demonstrate that the new two-channel DM-PCA method improves the group-level analyses of DWI data.

## 5. Discussion

One of the main contributions of our study is the development of an L1-norm based procedure for identifying a group of voxels with matched diffusion properties from noisy input data. Because of the high redundancy, across the diffusion-matched voxels, on their signal variation patterns along the diffusion dimension, noise in DWI data can be removed with DM-PCA without noticeably compromising anatomic resolvability.

In this study we have demonstrated the advantages of performing PCA denoising for real- and imaginary-components, in two separate channels, of complex-valued DWI data. Specifically, as shown by data in Figs 3 and 4, the complex-domain DM-PCA can much better suppress non-Gaussian signal induced bias in low-SNR data, as compared with magnitude-domain DM-PCA. The reduction of Rician and other types of non-Gaussian signal bias (e.g., in parallel DWI data) leads to a more accurate ADC fitting, as shown in Figs 3L and 4L. We have also shown that the complex-domain DM-PCA procedure can improve the statistical power in detecting the Parkinson’s disease related FA changes (see section 3.2).

It required about 2 min to denoise a 4D-DWI data set of 128 x 128 x 78 x 58 matrix size (in which 58 includes 8 baseline b = 0 images, and 25 DWI images with two repetitions: see section 2.3) using a Julia program on a PC (16GB memory; 4-core 2.7 GHz CPU). The data processing time may need to be further reduced (e.g., with parallel computation in general purpose GPU) for clinical uses in the future.

In addition to Fourier-domain and PCA based processing, it has been shown that DWI and DTI data can be denoised with other signal processing algorithms, ranging from wavelet-based smoothing [54], to the anisotropic diffusion filtering technique [55,56], to adaptive smoothing [57–59], to non-local means variants [60], to patch-based analysis [61], to rank constraints [62], to singular value decomposition [63], to sparseness and self-similarity based denoising [64]. These advanced methods were not evaluated in our current study.

Although our non-local PCA based denoising procedure is developed mainly for high-resolution DWI, this algorithm is expected to be applicable to other types of 4D MRI data, such as functional MRI, dynamic contrast enhanced imaging, and parametric mapping. Our lab is currently evaluating the performance of the non-local complex-domain PCA based denoising method on various types of non-DWI data.

In conclusion, in this study we report a two-channel complex-domain DM-PCA based denoising method that can effectively denoise high-resolution DWI data without noticeably compromising anatomic resolvability, when the SNR of input DWI data is 9 or higher. Our experimental results suggest that the DM-PCA denoising method performs reliably on human DWI and DTI data.
